# Novel pyridine bearing pentose moiety-based anticancer agents: design, synthesis, radioiodination and bioassessments

**DOI:** 10.1038/s41598-024-53228-4

**Published:** 2024-02-01

**Authors:** Marwa M. Mehany, Olfat A. Hammam, Adli A. Selim, Galal H. Sayed, Kurls E. Anwer

**Affiliations:** 1 Laboratory Department, Chemistry Unit, Police Hospital, Agouza, Cairo, Egypt; 2 Pathology Department, Theodor Bilharz Research Institute, Giza, Egypt; 3 Labeled Compounds Department, Hot Laboratories Centre, Egyptian Atomic Energy Authority (EAEA), Cairo, 13759 Egypt; 4 Heterocyclic Synthesis Lab., Chemistry Department, Faculty of Science, Ain Shams University, Abbassia, Cairo, 11566 Egypt

**Keywords:** Biochemistry, Cancer, Chemical biology, Drug discovery, Chemistry

## Abstract

Pyridine compounds are one of the most important heterocyclic derivatives showing wide ranges in biological and pharmacological activities. Green chemistry eliminates or reduces the generation of hazardous compounds. It prevents pollution at a molecular level. The microwave technique used in heterocyclic compound synthesis is also an important branch of green chemistry techniques. In this study, we report designing and synthesizing a new pyridine-bearing pentose moiety via a one-pot multicomponent reaction using D-glucose and also investigate its behavior and reactivity toward some simple and heterocyclic amino derivatives. The chemical structures of the synthesized compounds were characterized and tested for their cytotoxic activities. Some of the test compounds exhibited slight to high cytotoxic activities against Caco2 (colon cancer) cells, HepG2 (hepatocellular carcinoma) cells and MCF-7 (human breast cancer) cells by MTT assay. The results showed clearly that compound 4 and compound 8 displayed strongest to moderate cytotoxic activity against the HepG2, Caco2 and MCF-7 respectively and compound 1 showed good activity against MCF-7 in comparison to the standard anticancer drug doxorubicin. These data were by cytopathological examination. An in-vivo radioactive tracing study of compound 4 proved its targeting ability to sarcoma cells in a tumor-bearing mice model. Our findings suggest that the synthesized compounds may be promising candidates as novel anticancer agents.

## Introduction

Pyridine compounds are one of the most important heterocyclic derivatives widely used in petrochemical industry^1,2^, catalytic^3^ and polymer^4^ manufacturing. Heterocyclic chemicals, in particular pyridine, were applied in medicine and biology. The majority of pertinent medicinal compounds have been found to have pyridine scaffolds, offering a huge opportunity for therapeutic intervention^5^. Pyridines act on carbonic anhydrase inhibitors, this vital enzyme found in red blood cells, the mucosa of the stomach, pancreatic cells, and even the renal tubules. It sustains bone resorption, respiration, ureagenesis, gluconeogenesis, electrolyte secretion, and lipogenesis. It also maintains acid–base equilibrium. These processes involve CA isoenzymes, which are significant therapeutic targets that can be blocked to treat a range of illnesses, including cancer^6,7^. Liu and colleagues (2019) identified 2-amino-4-(1-piperidine) pyridine derivatives as new ALK/ROS1 dual inhibitors resistant to crizotinib^8^. Pyridines act as EGFR and HER-2 kinase inhibitors. EGFR family includes EGFR (HER1/ErbB-1), ErbB-2 (HER2/neu), ErbB-3 (HER3), and ErbB-4 (HER4). One gene linked to breast cancer is human epidermal growth factor receptor 2^9^. Overall pyridines function on a variety of targets, including topoisomerases, phosphoinositide 3-kinase, maternal embryonic leucine zipper kinase, C-met, EGFR, HER-2 kinase, CDK, PIM-1 kinase, and possible cytotoxic compounds^5^. Pyridine derivatives have partially harmful effects on the environment and humans and there are requirements for converting them into safe and useful products^10,11^. During the last decades, derivatives of pyridine showed a range of biological and pharmacological activities such as antagonist (for anti-inflammatory activity as p38α/MAPK14 inhibitor), anti-inflammatory, analgesic, herbicidal, anthelmintic, anticancer, antiviral, antioxidant, antimitotic, acaricidal, insecticidal and antimicrobial activities^12–20^. Furthermore, synthetic heterocyclic compounds containing nitrogen atoms have proven to have significant and diverse therapeutic potential. Thiazole, pyrazole, diazene and pyrimidine derivatives are reported to exhibit promising industry and biology applications ^21–25^. Carbohydrates are natural products that are considered environmentally sustainable compounds with a broad fascinating activity such as solubility in many polar solvents. This is because of the large number of OH groups thar are present in their molecule skeletons. Carbohydrate derivatives showed excellent therapeutic action against diabetes, cancer, HIV infection, etc. Also, their anti-inflammatory, antibiotics, antiviral, antimalarial and the properties of the glycosylation inhibitors. The heterocyclic derivatives from carbohydrates are openly used in our daily lives as detergents, cosmetics, clothes, food, lumber paper, sweetening agents, and so on. Furthermore, chemical transformations that involve using glucose or hexoses together with other industrially and biologically useful chemical compounds have become a top research point in the last decades. This is because glucose and/or hexoses are more superabundant and considered one of the most important natural renewable resources. Carbohydrates and their heterocyclic derivatives have an important place in different chemistry fields and their synthesis depends on microwaves are considered one of the most economical and versatile green techniques for the synthesis of many heterocyclic compounds^21^. Zhang et al. synthesized compounds containing pentose moiety and discovered that these compounds demonstrated a clear inhibitory effect on A450 lung cancer cells^26^. Green chemistry is the chemical process design science that eliminates or reduces hazardous compounds generation. It prevents pollution at a molecular level. The use of microwave technique in the synthesis of heterocyclic compounds is also an important branch of green chemistry techniques. One-pot multicomponent reaction^22^ is one of the most important tools for synthesis with facile execution, effectiveness for its productivity and highly diverse products generation in a single running and from easily starting materials. So, such techniques have much more attention because of their safety on the environment, improvement of the yield and time of the reaction and more convenient, and easily synthetic procedures which are highly energy efficient. Compared with microwave irradiation and conventional techniques are more environmentally tolerant, easily controlled, and friendly environmentally. As an advantage, many heterocyclic reactions were carried out in shorter reaction times, higher yields, and milder and cleaner conditions^27–30^. So now, this green synthesis type is considered a significant technique in heterocyclic chemistry synthesis because of its economy, simplicity, and mild conditions. The ongoing attempts to synthesize novel heterocycle derivatives are motivated by their previously successful uses in industry and biology^31–42^. In this study, we report designing and synthesizing a new pyridine-bearing pentose moiety via a one-pot multicomponent reaction using D-glucose^43–45^, and also investigate its behavior and reactivity toward some simple and heterocyclic amino derivatives. Also, the reactions were pressed using the thermal method along with the one-pot microwave technique. Furthermore, a comparison between the percentage yields and consumed times, which resulted from the two techniques was performed. The novel isolated products were illustrated by using different spectroscopic and analytical tools. The pharmacokinetic behavior of the most effective compound was studied with the aid of a radiolabeling technique to evaluate its targeting ability to tumor sites in the tumor-bearing mice model. The used radioisotope is iodine-131 which has a dual emission of both beta particles and gamma photons which makes its ability to be used as a theranostic agent^46–48^. The primary goal of this study was to design and synthesize new pyridine derivatives bearing pentose moiety for cancer-targeted chemo/radioisotope therapy.

## Experimental

### Materials and methods

All solvents, reagents and chemicals were bought from Sigma Aldrich. The used solvents were purified according to the standard methods. TLC was carried out on the plates of silica gel (Merck Kiesel gel 60F254, BDH) to monitor the progress of all synthesized compounds homogeneity and reactions. Microwave reactions were carried out with microwave reactor Anton Paar (monowave 300) using 10 mL borosilicate glass vials. All melting points were measured on a digital Stuart electric melting point apparatus “SMP3” and were uncorrected. Infrared spectra measurements (cm^−1^) were determined using KBr disks on a PerkinElmer 293 spectrophotometer. The ^1^H-NMR and ^13^C-NMR spectra were measured on a Varian Mercury 300 MHz spectrometer. All synthesized compounds were dissolved in DMSO–d_6_ as a solvent using tetramethyl silane as an internal standard. A GC-2010 Shimadzu Gas chromatography mass spectrometer (EI, 70 eV) was used for Mass spectrometry measurements. A Perkin-Elmer CHN-2400 analyzer was used for elemental microanalyses (CHN), the data were found to be in good agreement within ± 0.4% of the theoretical values. No-carrier-added [^131^I]NaI was received as a gift from RPF (Radioisotopes-Production-Facility), Egyptian Atomic Energy Authority (EAEA). A NaI (Tl) scintillation counter (Scaler Ratemeter SR7 model, the United Kingdom) was used for γ-ray radioactivity measurement.

### The reported compounds syntheses

The following sections will be given, the preparation of the starting material ethyl-6-amino-5-cyano-2-methyl-4-(1,2,3,4,5-pentahydroxypentyl)nicotinate (**1**), followed by compound **1** reaction procedures with some amino derivatives.

#### Ethyl-6-amino-5-cyano-2-methyl-4-((1S,2R,3R,4R)-1,2,3,4,5-pentahydroxypentyl) nicotinate (1)

A mixture of glucose (1.98 g, 10 mmol), malononitrile (0.66 g, 10 mmol), ethyl acetoacetate (1.30 mL, 10 mmol) and ammonium acetate (1.16 g, 15 mmol) in ethanol (10 mL) was refluxed for 2 h. The solid precipitated after cooling was collected by filtration, washed with ethanol/water (1:1) and crystallized from ethanol to form pale yellow crystals **1** (m.p 120–122 °C). IR (cm^−1^) ʋ: 3381 (OH), 3323, 3224 (NH_2_), 2218 (CN), 1721 (C=O), 1648 (C=N). ^1^H-NMR (300 MHz, DMSO-d_6_) δ (ppm): 1.19 (t, 3H, CH_3_CH_2_O, *J*=6 Hz), 2.38 (s, 3H, CH_3_-pyridine), 2.50–3.30 (broad, 5H, CH_2_ & 3CH-glucose), 4.12 (q, 2H, CH_3_CH_2_O, *J*=6.6 Hz), 4.69 (d, 1H, CH-glucose, *J*=3.4 Hz), 6.00–6.60 (m, 5H, 5OH, D_2_O Exchangeable), 8.88 (s, 2H, NH_2_, D_2_O exchangeable). ^13^C-NMR (300 MHz, DMSO-d_6_) δ (ppm): 14.1, 20.9, 42.9, 60.9, 63.6, 94.9, 115.3, 115.4, 120.0, 143.6, 143.7, 148.1, 152.8, 159.3 and 169.2. MS (m/z): 355 (M^+^, 31.30%). Anal. Calcd for C_15_H_21_N_3_O_7_ (355): C, 50.70; H, 5.92; N, 11.83. Found: C, 50.67; H, 6.04; N, 11.74.

#### General procedure for synthesis of compounds (2–12)

A solution of **1** (3.55 g, 10 mmol), one drop of concentrated sulphur acid in DMF (10 mL) was refluxed for 6–24 h with either aniline (0.93 mL, 10 mmol), 4-methoxy aniline (1.23 g, 10 mmol), 3-aminophenol (1.09 mL, 10 mmol), 4-aminoacetophenone (1.35 mL, 10 mmol), sulphaguanidine (2.14 g, 10 mmol), 4-aminoazobenzene (1.97 g, 10 mmol), 5-amino-2,3-dihydrophthalazine-1,4-dione (1.77 g, 10 mmol), 2-aminothiazole (1.00 g, 10 mmol), 4-amino-N-(thiazol-2-yl)benzenesulfonamide (2.55 g, 10 mmol), 4-amino-N-(pyrimidin-2-yl)benzenesulfonamide (2.50 g, 10 mmol), 4-aminoantipyrine (2.05 g, 10 mmol). After cooling, the mixture was poured onto cold ice water, the formed solid was collected by filtration, and recrystallized from proper solvent (supplementary file).

*(a) Ethyl-6-amino-5-cyano-2-methyl-4-((1R,2S,3S,4S)-1,2,3,4-tetrahydroxy-5-(phenylamino)pentyl)nicotinate* ***2***.

Yellow crystals from methanol (m.p. 180–182 °C). IR (cm^−1^) ʋ: 3382 (OH), 3324, 3225 (NH_2_), 3.099 (NH), 2218 (CN), 1721 (C=O), 1648 (C=N). ^1^H-NMR (300MHz, DMSO-d_6_) δ (ppm): 1.18 (t, 3H, CH_3_CH_2_O, *J*= 6.1 Hz), 2.50 (s, 3H, CH_3_-pyridine), 2.59 (s, 1H, CHNH-Ar), 3.15-3.36 (broad, 4H, CH_2_ & 2CH-glucose), 4.07 (q, 2H, CH_3_CH_2_O, *J* = 6.6 Hz), 4.83 (d, 1H, CH-glucose, *J* = 3.3 Hz), 5.72-5.95 (m, 4H, 4OH, D_2_O Exchangeable), 6.80–7.02 (m, 5H, Ar-H), 7.82 (s, 1H, NH, D_2_O Exchangeable), 8.50 (s, 2H, NH_2_, D_2_O Exchangeable). ^13^C-NMR (300MHz, DMSO-d_6_) δ (ppm): 13.8, 21.5, 55.2, 59.5, 60.3, 60.7, 60.8, 109.5, 111.0, 111.1, 114.9, 120.3, 123.5, 133.5, 138.2, 139.7, 144.8, 149.0, 165.5, and 168.5. MS (m/z): 430 (M^+^, 25.63%). Anal. Calcd for C_21_H_26_N_4_O_6_ (430): C, 58.60; H, 6.05; N, 13.02. Found: C, 58.74; H, 5.91; N, 12.99.

*(b) Ethyl-6-amino-5-cyano-2-methyl-4-((1R,2S,3S,4S)-1,2,3,4-tetrahydroxy-5-((4-methoxyphenyl)amino)pentyl) nicotinate* ***3***.

Brown crystals from methanol (m.p. 168–170 °C). IR (cm^−1^) ʋ: 3383 (OH), 3330, 3200 (NH_2_), 3103 (NH), 2220 (CN), 1722 (C=O), 1640 (C=N). ^1^H-NMR (300 MHz, DMSO-d_6_) δ (ppm): 1.21 (t, 3H, CH_3_CH_2_O, *J*=6 Hz), 2.50 (s, 3H, CH_3_-pyridine), 2.58 (s, 1H, CHNH-Ar), 3.21–3.30 (broad, 4H, CH_2_ & 2CH-glucose), 3.71 (s, 3H, OCH_3_), 4.08 (q, 2H, CH_3_CH_2_O, *J*=6.5 Hz), 4.78 (d, 1H, CH-glucose, *J*=3.4 Hz), 5.72–5.94 (m, 4H, 4OH, D_2_O Exchangeable), 6.83–7.35 (m, 4H, Ar–H), 8.33 (s, 1H, NH, D_2_O Exchangeable), 8.49 (s, 2H, NH_2_, D_2_O Exchangeable). ^13^C-NMR (300 MHz, DMSO-d_6_) δ (ppm): 14.1, 21.4, 55.1, 59.4, 60.4, 60.6, 60.8, 79.6, 109.5, 111.1, 111.4, 113.9, 115.5, 118.7, 119.9, 144.7, 146.4, 149.0, 152.9, 154.3, and 165.5. MS (m/z): 460 (M^+^, 25.70%). Anal. Calcd for C_22_H_28_N_4_O_7_ (460): C, 57.39; H, 6.09; N, 12.17. Found: C, 57.24; H, 6.19; N, 12.09.

*(c) Ethyl-6-amino-5-cyano-2-methyl-4-((1R,2S,3S,4S)-1,2,3,4-tetrahydroxy-5-((3-hydroxyphenyl)amino)pentyl) nicotinate* ***4***.

Beige crystals from acetone (m.p. 178–180 °C). IR (cm^−1^) ʋ: 3500–3100 (broad, OH & NH_2_), 2222 (CN), 1721 (C=O), 1650 (C=N). ^1^H-NMR (300 MHz, DMSO-d_6_) δ (ppm): 1.20 (t, 3H, CH_3_CH_2_O, *J*=6 Hz), 2.49 (s, 3H, CH_3_-pyridine), 2.58 (s, 1H, CHNH-Ar), 3.21–3.32 (broad, 4H, CH_2_ & 2CH-glucose), 4.07 (q, 2H, CH_3_CH_2_O, *J*=6.6 Hz), 4.62 (d, 1H, CH-glucose, *J*=3.5 Hz), 5.72–5.96 (m, 4H, 4OH, D_2_O Exchangeable), 6.19–7.00 (m, 4H, Ar–H), 7.69 (s, 1H, NH, D_2_O Exchangeable), 8.50 (s, 2H, NH_2_, D_2_O Exchangeable), 10.44 (s, 1H, OH, D_2_O Exchangeable). MS (m/z): 446 (M^+^, 27.96%). Anal. Calcd for C_21_H_26_N_4_O_7_ (446): C, 56.50; H, 5.83; N, 12.56. Found: C, 56.61; H, 5.89; N, 12.42.

*(d) Ethyl-4-((1R,2S,3S,4S)-5-((4-acetylphenyl)amino)-1,2,3,4-tetrahydroxypentyl)-6-amino-5-cyano-2-methylnicotinate* ***5***.

Yellow crystals from dioxane (m.p. 188–190 °C). IR (cm^−1^) ʋ: 3401 (OH), 3337, 3230 (NH_2_), 3100 (NH), 2222 (CN), 1721, 1680 (C=O), 1650 (C=N). ^1^H-NMR (300 MHz, DMSO-d_6_) δ (ppm): 1.19 (t, 3H, CH_3_CH_2_O, *J*=6 Hz), 2.51 (s, 6H, CH_3_CO & CH_3_-pyridine), 2.58 (s, 1H, CHNH-Ar), 3.19–3.44 (broad, 4H, CH_2_ & 2CH-glucose), 4.07 (q, 2H, CH_3_CH_2_O, *J*=6.6 Hz), 4.70 (d, 1H, CH-glucose, *J*=3.3 Hz), 5.72–5.96 (s, 4H, 4OH, D_2_O Exchangeable), 6.54–7.63 (m, 4H, Ar–H), 8.21 (s, 1H, NH, D_2_O Exchangeable), 8.50 (s, 2H, NH_2_, D_2_O Exchangeable). ^13^C-NMR (300 MHz, DMSO-d_6_) δ (ppm): 14.2, 21.5, 31.7, 46.1, 59.5, 60.2, 60.5, 60.8, 79.6, 109.5, 111.0, 111.1, 114.9, 118.3, 119.2, 144.8, 146.4, 149.0, 152.9, 154.3, 165.5 and 168.5. MS (m/z): 472 (M^+^, 41.50%). Anal. Calcd for C_23_H_28_N_4_O_7_ (472): C, 58.47; H, 5.93; N, 11.86. Found: C, 58.50; H, 6.03; N, 11.70.

*(e) Ethyl-6-amino-5-cyano-4-((1R,2S,3S,4S)-5-((4-(N-(diaminomethylene)sulfamoyl)phenyl)amino)-1,2,3,4-tetrahydroxypentyl)-2-methylnicotinate* ***6***.

Off white crystals from butanol (m.p. > 300 °C). IR (cm^−1^) ʋ: 3483–2500 (broad, OH & NH_2_), 2266 (CN), 1750–1550 (broad, C=O & C=N). ^1^H-NMR (300 MHz, DMSO-d_6_) δ (ppm): 1.19 (t, 3H, CH_3_CH_2_O, *J*=6 Hz), 2.38 (s, 3H, CH_3_-pyridine), 2.50–3.30 (broad, 5H, CH_2_ & 3CH-glucose), 4.12 (q, 2H, CH_3_CH_2_O, *J*=6.6 Hz), 4.69 (d, 1H, CH-glucose, *J*=3.4 Hz), 6.00–6.60 (m, 4H, 4OH, D_2_O Exchangeable), 6.72 (s, 4H, 2NH_2_, D_2_O exchangeable), 7.37–7.69 (m, 4H, Ar–H), 8.30 (s, 1H, NH, D_2_O Exchangeable),8.88 (s, 2H, NH_2_, D_2_O exchangeable). ^13^C-NMR (300 MHz, DMSO-d_6_) δ (ppm): 14.2, 21.3, 55.1, 59.5, 60.6, 60.0, 60.2, 79.5, 109.7, 111.0, 111.4, 114.1, 115.5, 118.7, 119.9, 144.7, 146.4, 149.0, 152.9, 154.3, and 165.5. MS (m/z): 551 (M^+^, 33.73%). Anal. Calcd for C_22_H_29_N_7_O_8_S (551): C, 47.91; H, 5.26; N, 17.79; S, 5.81. Found: C, 48.02; H, 5.15; N, 17.89; S, 5.72.

*(f) Ethyl-6-amino-5-cyano-2-methyl-4-((1R,2S,3S,4S)-1,2,3,4-tetrahydroxy-5-((4-((E)-phenyldiazenyl)phenyl)amino) pentyl)nicotinate (7)*.

Yellow crystals from dioxane (m.p. 176–178 ^O^C). IR (cm^−1^) ʋ: 3390 (OH), 3320, 3200 (NH_2_), 3080 (NH), 2219 (CN), 1721 (C=O), 1651 (C=N), 1450 (N=N). ^1^H-NMR (300 MHz, DMSO-d_6_) δ (ppm): 1.21 (t, 3H, CH_3_CH_2_O, *J*=6.1 Hz), 2.50 (s, 3H, CH_3_-pyridine), 2.57 (s, 1H, CHNH-Ar), 3.14–3.57 (broad, 4H, CH_2_ & 2CH-glucose), 4.07 (q, 2H, CH_3_CH_2_O, *J*=6.5 Hz), 4.76 (d, 1H, CH-glucose, *J*=3.4 Hz), 5.72–5.97 (m, 4H, 4OH, D_2_O Exchangeable), 6.66–7.89 (m, 9H, Ar–H), 8.20 (s, 1H, NH, D_2_O Exchangeable), MS (m/z): 534 (M^+^, 29.28%). Anal. Calcd for C_27_H_30_N_6_O_6_ (534): C, 60.67; H, 5.61; N, 15.73. Found: C, 60.55; H, 5.74; N, 15.71.

*(g) Ethyl-6-amino-5-cyano-4-((1R,2S,3S,4S)-5-((1,4-dioxo-1,2,3,4-tetrahydrophthalazin-5-yl)amino)-1,2,3,4-tetrahydroxypentyl)-2-methylnicotinate* ***8***.

Pale green crystals from dioxane (m.p. 200–202 °C). IR (cm^−1^) ʋ: 3451, 3420, 3382 (OH), 3324, 3223 (NH_2_), 3158, 3122 (NH), 2218 (CN), 1721, 1691, 1651 (C=O), 1600 (C=N). ^1^H-NMR (300 MHz, DMSO-d_6_) δ (ppm): 1.07 (t, 3H, CH_3_CH_2_O, *J*=6 Hz), 2.50 (s, 3H, CH_3_-pyridine), 2.58 (s, 1H, CHNH-Ar), 3.24–3.45 (broad, 4H, CH_2_ & 2CH-glucose), 4.05 (q, 2H, CH_3_CH_2_O, *J*=6.7 Hz), 4.76 (d, 1H, CH-glucose, *J*=3.3 Hz), 5.41(s, 1H, NH, D_2_O Exchangeable), 5.72–5.95 (m, 4H, 4OH, D_2_O Exchangeable), 6.87–7.47 (m, 5H, Ar–H & NH_2_, D_2_O Exchangeable), 11.16 (broad, 2H, 2NH, D_2_O Exchangeable). ^13^C-NMR (300 MHz, DMSO-d_6_) δ (ppm): 14.1, 21.4, 59.4, 60.3, 60.5, 60.7, 79.6, 109.4, 116.3, 118.7, 120.6, 126.5, 127.1, 133.8, 142.8, 144.7, 146.4, 148.9, 150.6, 151.4, 161.3, 156.4, and 168.4. MS (m/z): 514 (M^+^, 42.07%). Anal. Calcd for C_23_H_26_N_6_O_8_ (514): C, 53.70; H, 5.06; N, 16.34. Found: C, 53.79; H, 4.98; N, 16.29.

*(h) Ethyl-6-amino-5-cyano-2-methyl-4-((1R,2S,3S,4S)-1,2,3,4-tetrahydroxy-5-(thiazol-2-ylamino)pentyl)nicotinate* ***9***.

Pale brown crystals from butanol (m.p. 222–224 °C). IR (cm^−1^) ʋ: 3381 (OH), 3324, 3225 (NH_2_), 3102 (NH), 2220 (CN), 1721 (C=O), 1648, 1600 (C=N). ^1^H-NMR (300 MHz, DMSO-d_6_) δ (ppm): 1.20 (t, 3H, CH_3_CH_2_O, *J*=6 Hz), 2.50 (s, 3H, CH_3_-pyridine), 2.58 (s, 1H, CHNH-Ar), 3.18–3.40 (broad, 4H, CH_2_ & 2CH-glucose), 4.07 (q, 2H, CH_3_CH_2_O, *J*=6.6 Hz), 4.72 (d, 1H, CH-glucose, *J*=3.4 Hz), 5.72–5.94 (m, 4H, 4OH, D_2_O Exchangeable), 6.92–7.21 (m, 2H, Ar–H), 8.12(s, 1H, NH, D_2_O Exchangeable), 8.50 (s, 2H, NH_2_, D_2_O Exchangeable). MS (m/z): 437 (M^+^, 35.53%). Anal. Calcd for C_18_H_23_N_5_O_6_S (437): C, 49.43; H, 5.26; N, 16.02; S, 7.32. Found: C, 49.360; H, 5.30; N, 15.91; S, 7.41.

*(i) Ethyl-6-amino-5-cyano-2-methyl-4-((1R,2S,3S,4S)-1,2,3,4-tetrahydroxy-5-((4-(N-(thiazol-2-yl)sulfamoyl)phenyl) amino)pentyl)nicotinate* (***10***).

Off white crystals from acetone (m.p. 178–180 °C). IR (cm^−1^) ʋ: 3384, 3324 (OH), 3322, 3225 (NH_2_), 3263 3090 (NH), 2219 (CN), 1721 (C=O), 1648 (C=N)^1^H-NMR (300 MHz, DMSO-d_6_) δ (ppm): 1.20 (t, 3H, CH_3_CH_2_O, *J*=6 Hz), 2.54 (s, 3H, CH_3_-pyridine), 2.63 (s, 1H, CHNH-Ar), 3.22–3.48 (broad, 4H, CH_2_ & 2CH-glucose), 4.07 (q, 2H, CH_3_CH_2_O, *J*=6.5 Hz), 4.76 (d, 1H, CH-glucose, *J*=3.4 Hz), 5.73–5.94 (m, 4H, 4OH, D_2_O Exchangeable), 5.77 (s, 1H, NH, D_2_O Exchangeable), 6.55–7.46 (m, 6H, Ar–H), 8.48 (s, 2H, NH_2_, D_2_O Exchangeable), 12.39 (s, 1H, NH, D_2_O Exchangeable). MS (m/z): 592 (M + , 4%). Anal. Calcd for C_24_H_28_N_6_O_8_S_2_ (592): C, 48.65; H, 4.73; N, 14.19; S, 10.81. Found: 48.59; H, 4.82; N, 14.21; S, 10.77.

*(j) Ethyl-6-amino-5-cyano-2-methyl-4-((1R,2S,3S,4S)-1,2,3,4-tetrahydroxy-5-((4-(N-(pyrimidin-2-yl)sulfamoyl) phenyl)amino)pentyl)nicotinate* (***11***).

White crystals from acetone (m.p. 220–222 °C C). IR (cm^−1^) ʋ: 3423, 3361, 3354 (OH), 3322, 3225 (NH_2_), 3102, 3074 (NH), 2218 (CN), 1720 (C=O), 1649 (C=N). 1H-NMR (300 MHz, DMSO-d6) δ (ppm): 1.19 (t, 3H, CH_3_CH_2_O, *J*=6 Hz), 2.50 (s, 3H, CH_3_-pyridine), 2.59 (s, 1H, CHNH-Ar), 3.22–3.36 (broad, 4H, CH_2_ & 2CH-glucose), 4.07 (q, 2H, CH_3_CH_2_O, *J*=6.6 Hz), 4.76 (d, 1H, CH-glucose, *J*=3.2 Hz), 5.74–5.93 (m, 4H, 4OH, D_2_O Exchangeable), 5.96 (s, 1H, NH, D_2_O Exchangeable), 6.57–8.48 (m, 9H, Ar–H & NH_2_, D_2_O Exchangeable), 11.23 (s, 1H, NH, D_2_O Exchangeable). ^13^C-NMR (300 MHz, DMSO-d6) δ (ppm): 14.2, 21.6, 59.5, 60.8, 79.6, 109.5, 111.0, 111.2, 112.2, 115.0, 115.6, 124.9, 129.9, 144.9, 19.1, 153.1, 157.3, 158.3, 165.6, and 168.5. MS (m/z): 587 (M^+^, 2.7%). Anal. Calcd for C_25_H_29_N_7_O_8_S (587): C, 51.11 H, 4.94; N, 16.70; S, 5.45. Found: C, 51.04 H, 5.06; N, 16.61; S, 5.47.

*(k) Ethyl-6-amino-5-cyano-4-((1R,2S,3S,4S)-5-((1,5-dimethyl-3-oxo-2-phenyl-2,3-dihydro-1H-pyrazol-4-yl)amino)-1,2,3,4-tetrahydroxypentyl)-2-methylnicotinate * (***12***).

Orange crystals from dioxane (m.p. 176–178 °C). IR (cm^−1^) ʋ: 3400 (OH), 3334, 3200 (NH_2_), 3090 (NH), 2222 (CN), 1721, 1671 (C=O), 1648, 1620 (C=N). ^1^H-NMR (300 MHz, DMSO-d6) δ (ppm): 1.19 (t, 3H, CH_3_CH_2_O, *J*=6. Hz), 2.23 (s, 3H, CH_3_-C=C), 2.51 (s, 3H, CH_3_-pyridine), 2.57 (s, 1H, CHNH-Ar), 3.24–3.45 (broad, 7H, CH_2_, 2CH-glucose &CH_3_-N), 4.07 (q, 2H, CH_3_CH_2_O, *J*=6.5 Hz), 4.88 (d, 1H, CH-glucose, *J*=3.2 Hz), 5.72–5.95 (m, 4H, 4OH, D_2_O Exchangeable), 7.00–7.47 (m, 5H, Ar–H), ), 8.50 (s, 2H, NH_2_, D_2_O Exchangeable), 10.86 (broad, 1H, NH, D_2_O Exchangeable). ^13^C-NMR (300 MHz, DMSO-d6) δ (ppm): 12.6, 14.1, 21.4, 35.9, 59.4, 59.9, 60.3, 60.5, 60.7, 79.6, 109.4, 116.3, 117.2, 118.7, 128.3, 133.4, 140.0, 144.1, 146.2, 148.9, 154.6, 155.2, 160.4, 163.3, 167.9, and 170.7. MS (m/z): 540 (M^+^, 17.81%). Anal. Calcd for C_26_H_32_N_6_O_7_ (540): C, 57.78 H, 5.93; N, 15.56. Found: C, 57.60 H, 6.01; N, 15.51.

### Comparison between microwave and thermal methods

In the microwave reactions, the amount of the same reactants in the thermal technique was used. The reaction completion was illustrated by using TLC. The reaction mixtures were washed with ethanol and crystallized from a suitable solvent. The thermal and microwave reaction times are shown in (Table 1). Comparisons in terms of yields and times between the prepared compounds by using thermal and microwave techniques were reported. However, we used the yield economy (YE) as a term to determine the thermal and microwave synthetic different efficiencies of the same reaction. Calculation of YE was occurred through: $YE= \frac{yield{\%}}{Reaction~ time~ "min"}$. In this report, the YE was used to provide the yields obtained conclusively enhanced under microwave and thermal conditions. The equation of RME is: $\mathrm{RME }=\frac{Wt~ of~ isolateted ~product }{Wt~ of~ reactants}.$While, OE was used for the direct comparisons between the two reaction types and can be calculated through $OE= \frac{RME}{AE} \times100$. So, we can consider the yield economy (YE) as a metric to enhance the conversion efficiencies of these two different synthetic methods of the same reaction. The reaction's theoretical maximum efficiency was represented by using AE, while RME gives the observed mass efficiency. The thermal and microwave reactions atomic economy (AE) have the same values due to using two different reaction conditions to obtain the same desired compounds, as shown in (Table 1).

**Table 1:** Show the comparison in terms of physical data between the synthesized compounds under thermal and microwave techniques.

Cpd. no	Time “min”		Yield %		YE		RME		OE		AE
	Th	M.W	Th	M.W	Th	M.W	Th	M.W	Th	M.W	
1	120	2	52	95	0.4333	47.50	32.39	59.17	46.53	85.00	69.61
2	720	12	55	96	0.0764	8	38.21	66.69	48.52	84.69	78.75
3	600	10	58	90	0.0967	9	41.11	63.79	52.13	80.89	78.86
4	960	11	53	92	0.0552	8.36	37.23	64.62	46.91	81.43	79.36
5	1200	12	58	95	0.0483	7.92	49.63	67.84	61.83	84.51	80.27
6	1440	14	56	94	0.0389	6.71	41.70	69.99	50.48	84.72	82.61
7	1200	11	55	96	0.0458	8.73	40.62	70.90	49.45	86.31	82.15
8	1140	10	57	97	0.0500	9.70	41.68	70.92	51.08	86.92	81.59
9	1080	9	58	89	0.0537	9.89	40.49	62.13	51.24	78.63	79.02
10	960	8	59	93	0.0615	11.63	44.72	70.49	53.48	84.30	83.62
11	600	8	55	94	0.0917	11.75	41.60	71.11	49.82	85.16	83.50
12	360	4	53	96	0.1472	24	39.15	70.92	47.70	86.41	82.07

### Cytotoxicity assay

Three human tumour cell lines namely, Hepatocellular carcinoma (HepG2), Mammary gland breast cancer (MCF-7) and Colorectal adenocarcinoma (Caco-2). The cell lines were obtained from ATCC via Holding company for biological products and vaccines (VACSERA), Cairo, Egypt. Doxorubicin was used as a standard anticancer drug for comparison. The reagents RPMI-1640 medium, MTT and DMSO (sigma Co., St. Louis, USA), Fetal Bovine serum (GIBCO, UK).

*MTT assay:* The cell lines mentioned above were used to determine the inhibitory effects of compounds on cell growth using the MTT assay. This colorimetric assay is based on the conversion of the yellow tetrazolium bromide (MTT) to a purple formazan derivative by mitochondrial succinate dehydrogenase in viable cells. Cell lines were cultured in RPMI-1640 medium with 10% fetal bovine serum. Antibiotics added were 100 units/ml penicillin and 100 µg/ml streptomycin at 37 C in a 5% Co2 incubator. The cell lines were seeded in a 96-well plate at a density of 1.0 × 10^4^ cells/well. at 37 C for 48 h under 5% Co2. After incubation, the cells were treated with different concentrations of compounds and incubated for 24 h. After 24 h of drug treatment, 20 µl of MTT solution at 5 mg/ml was added and incubated for 4 h. Dimethyl sulfoxide (DMSO) in volume of 100 µl was added into each well to dissolve the purple formazan formed. The colorimetric assay was measured and recorded at the absorbance of 570 nm using a plate reader (EXL 800, USA). The relative cell viability in percentage was calculated as (A570 of treated samples/A570 of untreated sample) X 100 ^43^ (Table 2).

**Table 2:** Anticancer activity of the synthesized compounds against HepG2, Caco-2 and MCF-7 cell lines.

Compound No	In-vitro Cytotoxicity IC50 (µM)*		
	HepG2	Caco-2	MCF-7
DOX	4.50 ± 0.2	12.49 ± 1.1	4.17 ± 0.2
1	27.03 ± 1.9	46.32 ± 2.6	19.17 ± 1.4
2	70.67 ± 3.4	64.05 ± 3.5	53.46 ± 3.1
3	63.48 ± 3.3	83.50 ± 3.9	48.75 ± 2.8
4	11.78 ± 0.9	14.81 ± 1.2	8.76 ± 0.7
5	86.23 ± 4.0	> 100	73.52 ± 3.8
6	55.64 ± 3.1	52.06 ± 2.9	68.75 ± 3.6
7	42.91 ± 2.6	35.96 ± 2.3	38.49 ± 2.4
8	18.53 ± 1.3	23.11 ± 1.8	13.25 ± 1.1
10	36.20 ± 2.4	29.87 ± 2.1	24.82 ± 1.8
11	49.65 ± 2.9	58.42 ± 3.2	41.30 ± 2.6
12	74.95 ± 3.8	92.24 ± 4.6	57.18 ± 3.4

*IC50 (µM): 1–10 (very strong). 11–20 (strong). 21– 50 (moderate). 51–100 (weak) and above 100 (non-cytotoxic).

*DOX* Doxorubicin.

It was noted that compounds 4 and 8 have stronger cytotoxic activities than other compounds with values of 11.7 and 18.53, respectively, because they have hydrophilic and lipophilic parts that compound 4 has a hydroxyl group which increased the solubility of the compound due to ionic group and compound 8 have amine group which is lipophilic and hydrophilic which can interact with water through hydrogen bond.

### Structure activity relationship

**Figure 1:**
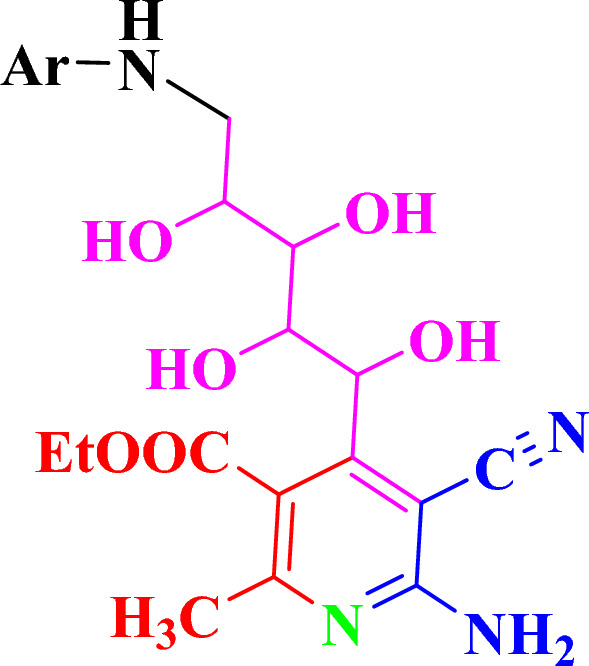
Main nucleus for the synthesized derivatives.

In the current report, a series of (b) Ethyl-6-amino-5-cyano-2-methyl-4-((1R,2S,3S,4S)-1,2,3,4-tetrahydroxy-5-((Aryl)amino)pentyl) nicotinate derivatives were prepared and tested their cytotoxicity on three cancer cell lines Caco2, HepG2 and MCF-7. Various derivatives were utilized on the position of 5 amino pentyl moiety at Ar (Fig. 1). First, a 3-hydroxyphenyl substitution Ar=3-OH–C_6_H_4_-forming an 5-((3-hydroxyphenyl)amino)pentyl moiety appeared to give the highest cytotoxic activity. Furthermore, modulations were performed where Ar was a different aromatic and heterocyclic groups which showed a strong decrease in activities. While a 1,4-dioxo-1,2,3,4-tetrahydrophthalazin-5-yl moiety compound 8 caused a moderated cytotoxic activity effect. However, a 4-(N-(thiazol-2-yl)sulfamoyl)phenyl compound 10 showed a continuously dramatic cytotoxic activity decrease. Moreover, modulations of the Aryl substitution using phenyl, 4-methoxyphenyl, 4-(N-(diaminomethylene)sulfamoyl)phenyl, 4-((E)-phenyldiazenyl)phenyl, 4-(N-(pyrimidin-2-yl)sulfamoyl) phenyl and 1,5-dimethyl-3-oxo-2-phenyl-2,3-dihydro-1H-pyrazol-4-yl caused further cytotoxic activity decreasing. Finally, using 4-acetylphenyl as elucidated in compound 5 displayed the lowest cytotoxic activity through the studied series.

### Radiosynthesis and in-vivo biodistribution of radioiodinated compound 4

#### Synthesis of radioiodinated compound 4

Radioactive iodine (I-131) was electrophilically introduced in compound 4 by using chloramine-T which oxidize [^131^I]iodide to [^131^I]iodonium form^49,50^. The radiolabeling process was carried out through the optimization of different parameters: Chloramine-T: from 100 to 600 µg, pH: from 4 to 9, Compound 4 content: from 100 to 600 µg, and Reaction time: from 15 min to 24 h. Radioiodinated compound 4 formation was calculated by chromatographic technique using paper chromatography and TLC to evaluate the highest radiochemical purity.

#### In-vivo studies of the radioiodinated compound 4 in sarcoma-bearing mice

### Ethical statement

All animal procedures and experimental protocols were performed following the International Guiding Principles for Biomedical Research Involving Animals ^51^ and were approved by the research ethics committee, Ain Sams University, Egypt (code: ASU-SCI/CHEM/2023/4/1). The current study adheres to the ARRIVE guidelines for reporting in-vivo experiments^52^.

Sarcoma was induced in mice model using the Ehrlich cell line in the right thigh muscle by intramuscularly injecting 100 µL of the cell line suspension (12.5 × 10^6^ cells ml^–1^)^53^. Female Swiss albino mice were collected in five groups (20–25 g). About 50 µL radioiodinated-compound 4 containing about 5 MBq were intravenously injected. After the pre-determined time, mice were anesthetized using isoflurane, then they were dissected. The body organs of interest and fluids were collected and weighed. The radioactivity of each organ was counted using a NaI (Tl) crystal gamma counter. Bone, blood, and muscles were evaluated as 10, 7, and 40% of the body weight, respectively^54^. % Injected dose per gram (% ID/gram ± S.D.) was calculated at each time point for each group.

## Results and discussion

### Synthesis

**Scheme 1:**
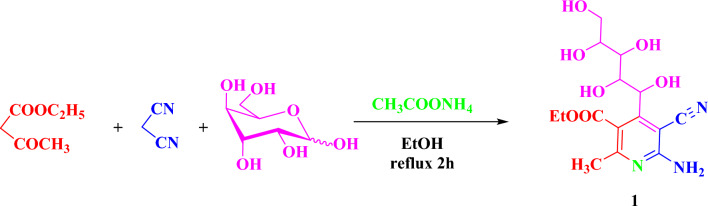
Formation of ethy16-amino-5-cyano-2-methy1-4-(1,2,3,4,5-pentahydroxypentyl) nicotinate, 1.

The ethyl-6-amino-5-cyano-2-methyl-4-((1S,2R,3R,4R)-1,2,3,4,5-pentahydroxypentyl) nicotinate (1) was synthesized through condensation of D-glucose, malononitrile, ethyl acetoacetate and ammonium acetate in ethanol via a new one-pot four component methodology (Scheme 1). The structure of compound **1** was illustrated using mass spectrum which showed a molecular ion peak at m/z=355 (M^+^, 31.30%) corresponding to molecular formula C_15_H_21_N_3_O_7_.

**Scheme 2:**
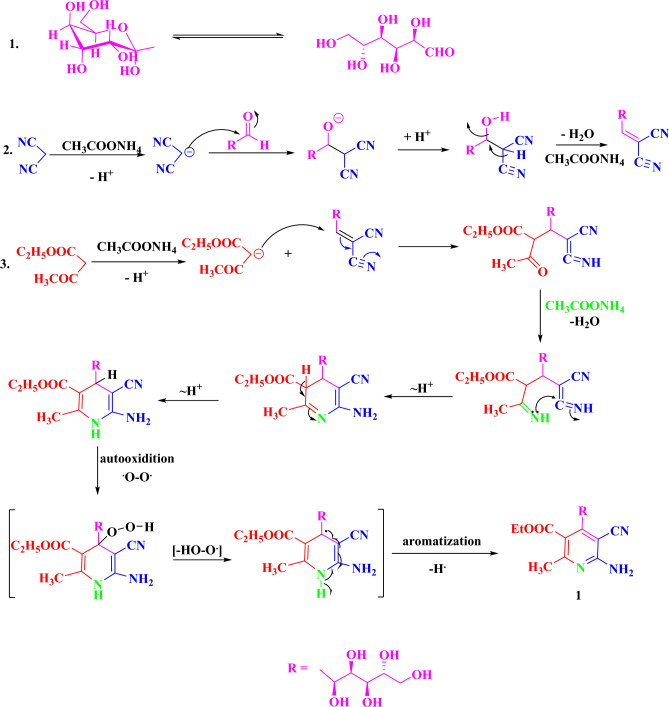
Mechanism of formation of pyridine derivative, 1.

Compound **1** probably formed through the supposed mechanism shown in (Scheme 2). Compound **1** may occur firstly through a nucleophilic attack from ammonium acetate on malononitrile with the removal of one acidic hydrogen-formed carbanion, which attacks the C=O of the D-glucose followed by removing one molecule of water to give Schiff base. Secondary nucleophilic attack from ammonium acetate on ethyl acetoacetate with the removal of one acidic hydrogen formed carbanion, which makes 1.4-addition with the Schiff base followed by cyclization formed 1,4-dihydroryidine. Finally, occur aromatization by autoxidation at room temperature. Also, Compound 1 probably formed through the supposed mechanism shown in (Scheme 2). Proton transfer from the N-atom on the 1,4-dihydropyridine ring initiates the aromatization of Hantzsch dihydropyridines by superoxide. This anion dihydropyridine then easily passes through additional homogenous oxidations to provide the final aromatized products^55–57^.

**Scheme 3:**
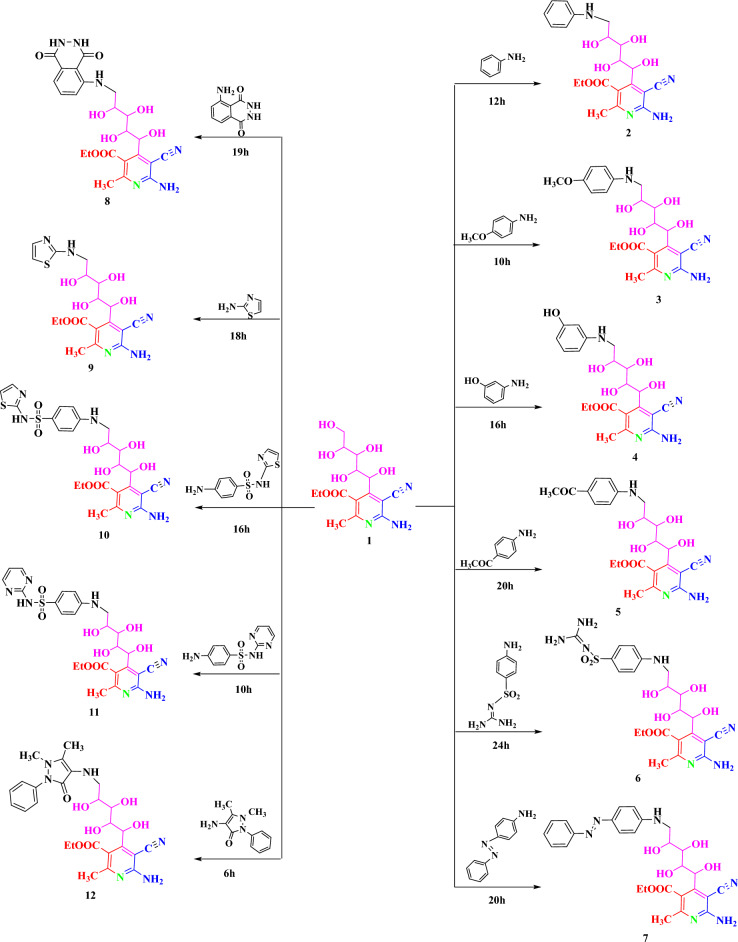
Formation of compounds 2–12.

Reactions of compound **1** toward nitrogen nucleophiles such as aromatic and heterocyclic amines namely aniline, 4-methoxy aniline, 3-aminophenol, 4-aminoacetophenone, sulphaguanidine, 4-aminoazobenzene, 5-amino-2,3-dihydrophthalazine-1,4-dione, 2-aminothiazole, 4-amino-N-(thiazol-2-yl)benzenesulfonamide, 4-amino-N-(pyrimidin-2-yl)benzenesulfonamide, 4-aminoantipyrine have been investigated and produced the corresponding condensed compounds **2–12**, which were expected to have some interesting biological activities (Scheme 3).

**Scheme 4.**
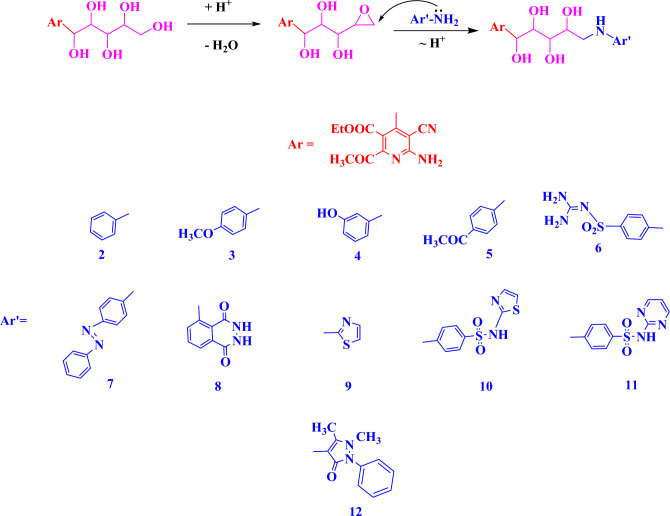
Mechanism of formation of compounds 2–12.

Compounds 2–12 probably formed through the supposed mechanism shown in (Scheme 4).

### Cytopathological examinations

Cultured breast cancer (MCF-7), colon cancer (Caco-2) and hepatocellular carcinoma (HepG2). The cell lines were obtained from ATCC via Holding company for biological products and vaccines (VACSERA), Cairo, Egypt, cells were trypsinized, washed in PBS, pH=7.4 and collected in a tube. The samples were centrifuged at a rate of 1200–1500 r/min for 15 min using Shandon Cytospin (Thermo Fisher Scientific, Waltham, Massachusetts). The cell pellet was spread on glass slides. Slides were immediately fixed in 95% ethanol for 24 h. The slides were stained with hematoxylin and eosin (H & E).

**Figure 2:**
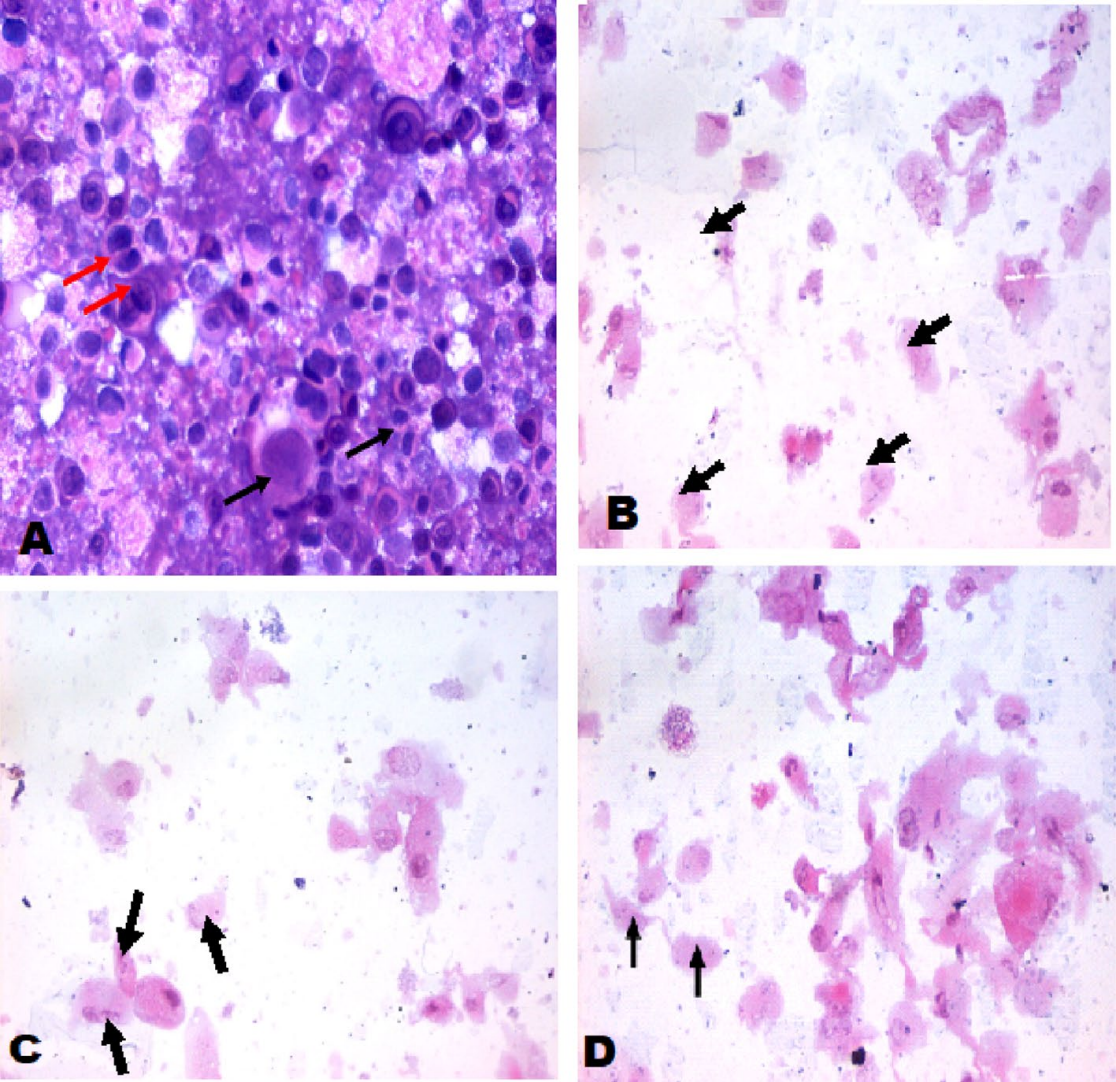
Cytospin smear stained by H & E (400×). (**A**) Untreated colon carcinoma cell line (Caco-2) cell, (**B**) (Caco-2) cells treated with the Doxorubicin was used as a standard anticancer drug, (**C**) Caco-2) cells treated with the compound 4, (**D**) Caco-2) cells treated with the compound 8.

**Figure 3:**
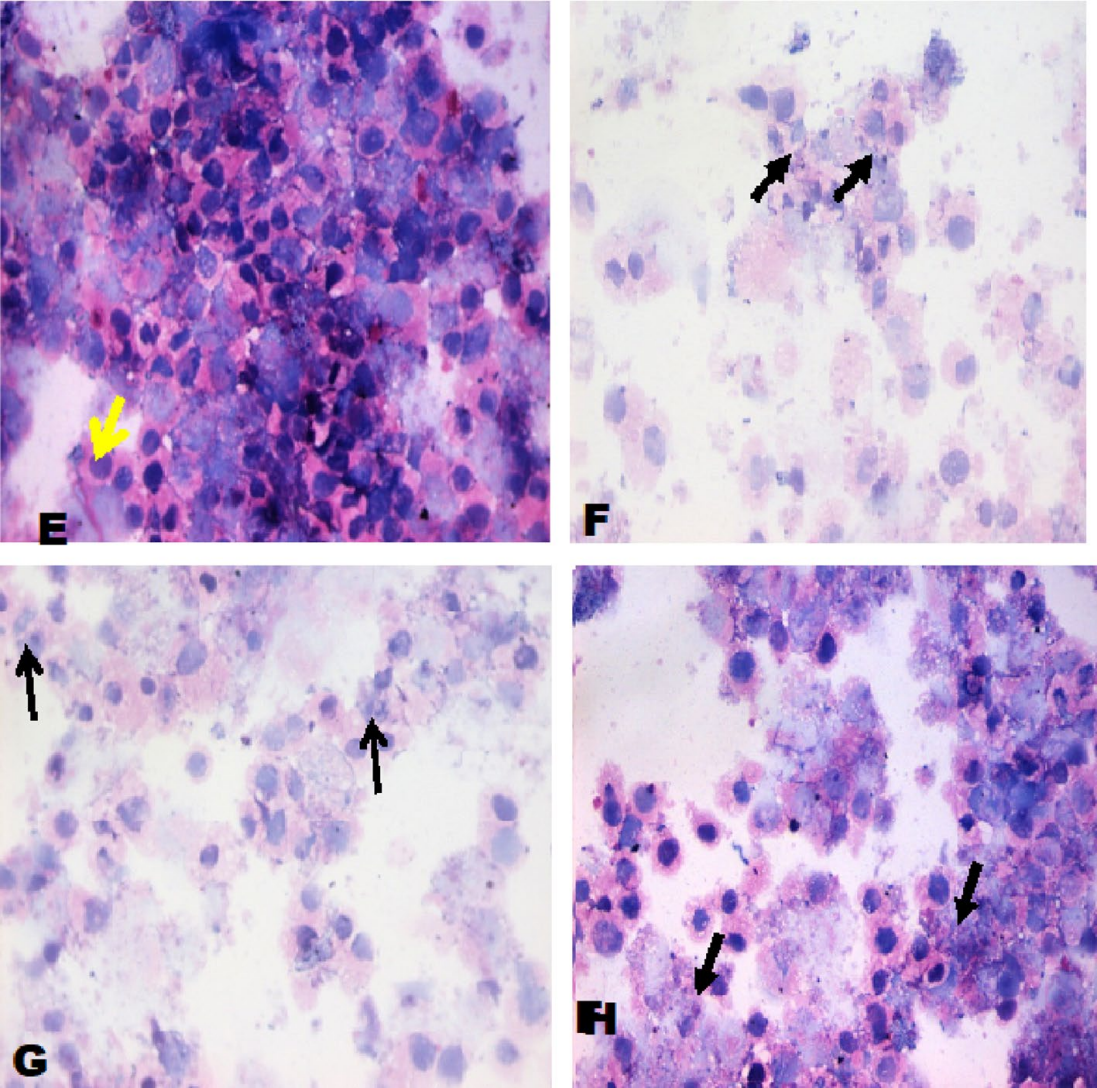
Cytospin smear stained by H & E (400 ×). E- Untreated hepatocellular carcinoma (HepG2) cells F- HepG2 cells treated with the Doxorubicin was used as a standard anticancer drug, G- HepG2 cells treated with the compound 4, H: HepG2 cells treated with the compound 8.

**Figure 4:**
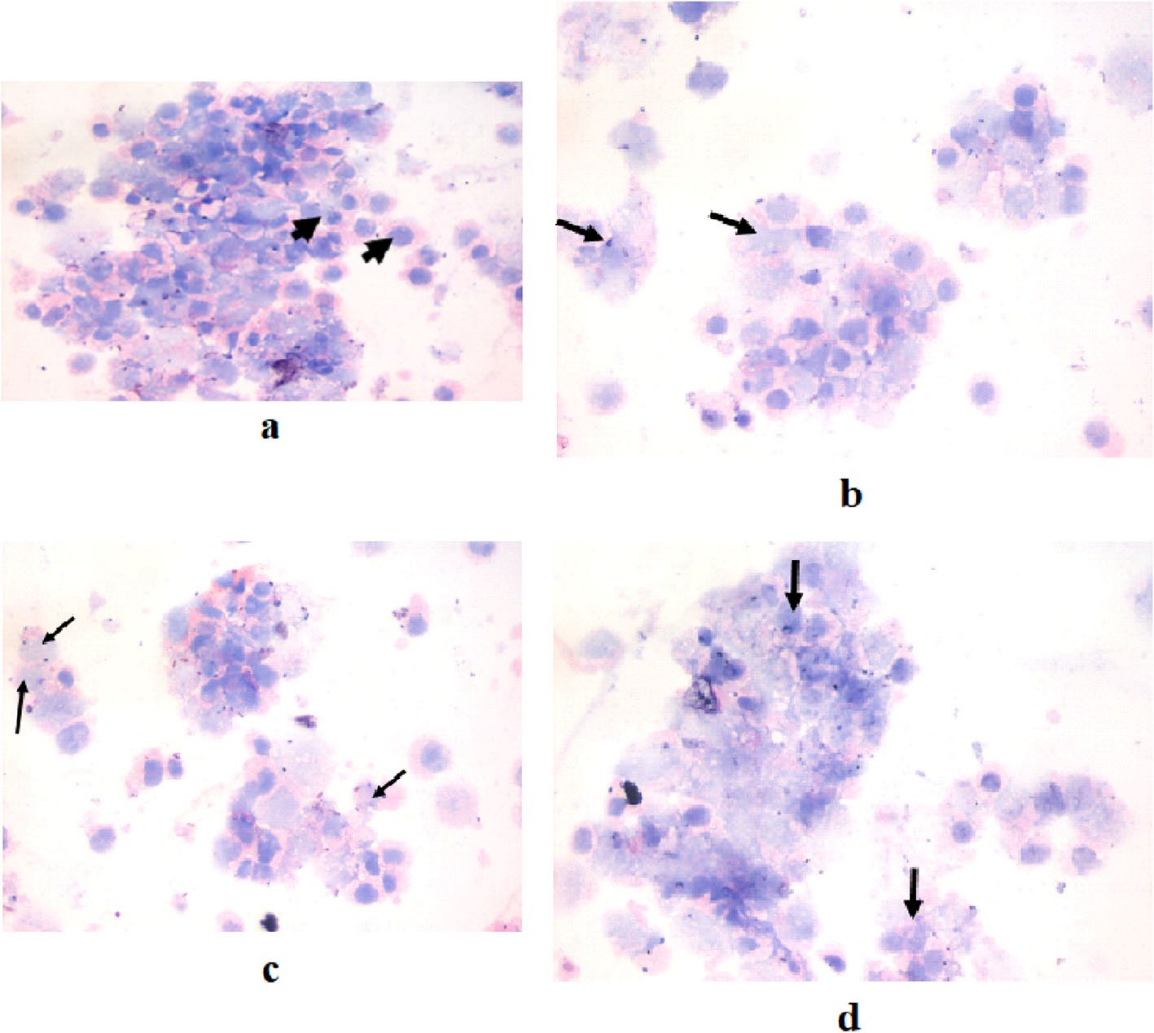
Cytospin smear stained by H & E (400 ×). A- Untreated Mammary gland breast cancer (MCF-7) cells, b- Mammary gland breast cancer (MCF-7) treated with the Doxorubicin was used as a standard anticancer drug, c- Mammary gland breast cancer (MCF-7) cells treated with the compound 4, d- Mammary gland breast cancer (MCF-7) treated with the compound 8.

It was noted from Figs. 2, 3 and 4 that cells treated with compounds 4 and 8 showed a moderate number of cancer cells with central nuclei (black arrow) and with scattered apoptotic and degenerative changes compared with control (untreated cell) which have high nucleocytoplasmic ratio and showing a large number of neoplastic cancer cell consisting of groups of epithelial cells with enlarged nuclei.

### Radiosynthesis and in-vivo studies of radioiodinated compound 4

The ADME parameters of the synthesized compounds were intensively studied using the field of radiopharmaceutical chemistry^53^. Due to radioiodine's high compatibility with a variety of organic compounds and simple physical radio-imaging screening, radioiodine is particularly successful at radioiodinating organic compounds to act as a screening probe of their in-vivo biodistribution pattern^53^. Considering that among all the investigated compounds, compound 4 showed the highest cytotoxicity. So, it was chosen for investigations on radiolabeling and biodistribution.

#### Radiosynthesis of radioiodinated compound 4

**Figure 5:**
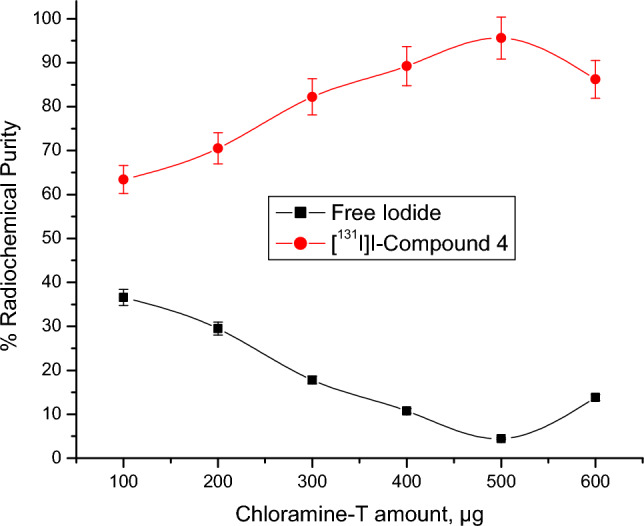
Impact of CAT amount on RCP of [^131^I]I-compound 4. Conditions for the reaction: 400 µg of compound 4, pH 8, and 5 µl of [^131^I] NaI solution (3.7 MBq) after 30 min at room temperature, N=5 separate tests.

**Figure 6:**
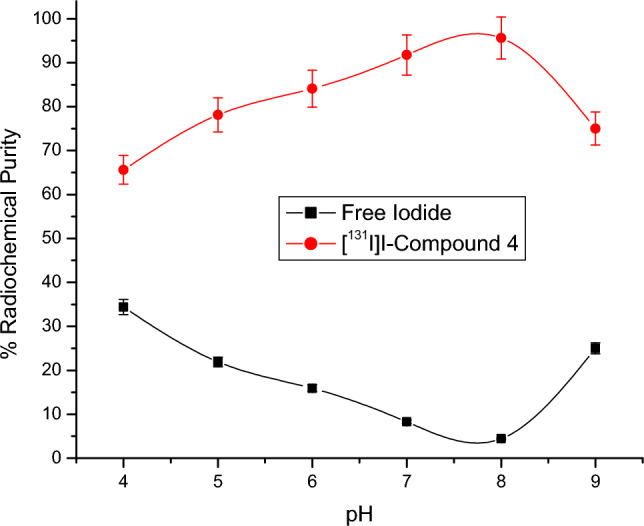
Impact of pH on RCP of [^131^I]I-compound 4. Conditions for the reaction: 500 µg of CAT, 400 µg of compound 4, and 5 µl of [^131^I] NaI solution (3.7 MBq) after 30 min at room temperature, N=5 separate tests.

**Figure 7:**
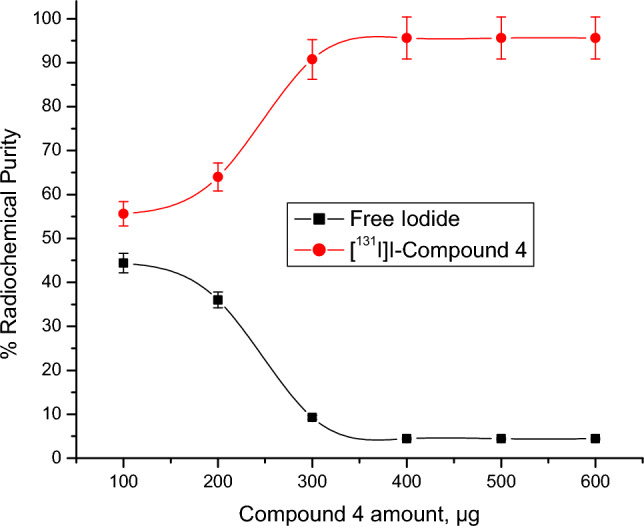
Impact of compound 4 amount on RCP of [^131^I]I-compound 4. Conditions for the reaction: 500 µg of CAT, pH 8 and 5 µl of [^131^I] NaI solution (3.7 MBq) after 30 min at room temperature, N=5 separate tests.

**Figure 8:**
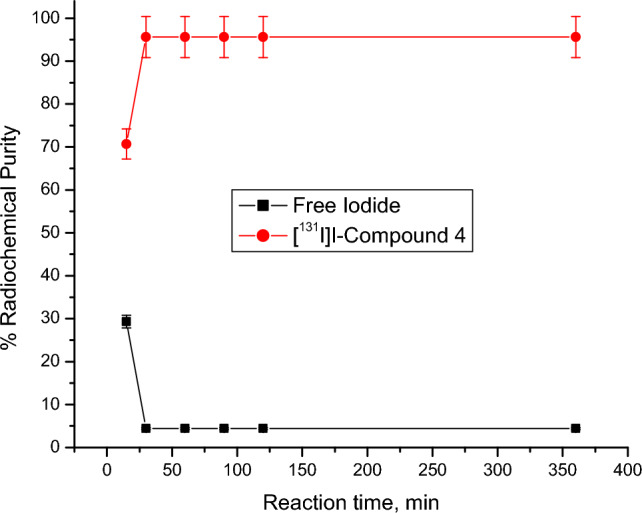
Impact of reaction time on RCP of [^131^I]I-compound 4. Conditions for the reaction: 500 µg of CAT, 400 µg of compound 4, pH 8 and 5 µl of [^131^I] NaI solution (3.7 MBq) at room temperature, N=5 separate tests.

Compound 4 was radiolabeled with iodine-131 to assess its pharmacokinetics and to study its ability to deliver the therapeutic radioisotope (iodine-131) to the target site (cancer) for radioisotope-targeted therapy. The highest radiochemical purity of radioiodinated compound 4 was achieved via the optimization of all variables. Iodine-131 was electrophilically substituted while being exposed to an oxidizing agent (chloramine-T). The radioiodination process is significantly impacted by chloramine-T, which transforms iodide ions into iodonium ions and also allows an electrophilic substitution to take place^58^. Figure 5 demonstrates that the best dose of 400 µg of CAT results in the highest RCP of 95.59%. Low or high levels of CAT may be the cause of insufficient oxidation of I-131 and unwanted oxidative byproduct^59^. The pH levels of the reaction mixture had a big impact on RCP. The RCP was increased by raising pH from acidic to almost neutral levels (pH 8) and decreased by lowering pH levels by more than 8 (Fig. 6). This drop in RCP may be caused by the creation of iodate (IO_3_^-^) and hypoiodite (IO^-^) ions^60^. Figure 7 illustrates the influence of compound 4 (substrate) amount on the RCP. The maximum RCP was obtained at 100 µg of substrate, which may capture every iodonium ion from the reaction mixture. There are no appreciable variations in RCP as substrate amounts are increased. Figure 8 depicts the speed of this reaction and the stability of the radioiodinated molecule that resulted. After 30 min, the reaction was finished, and a 24-h stability study was conducted. (supplementary file).

#### In-vivo studies of radioiodinated compound 4 in a sarcoma-bearing mice

**Figure 9:**
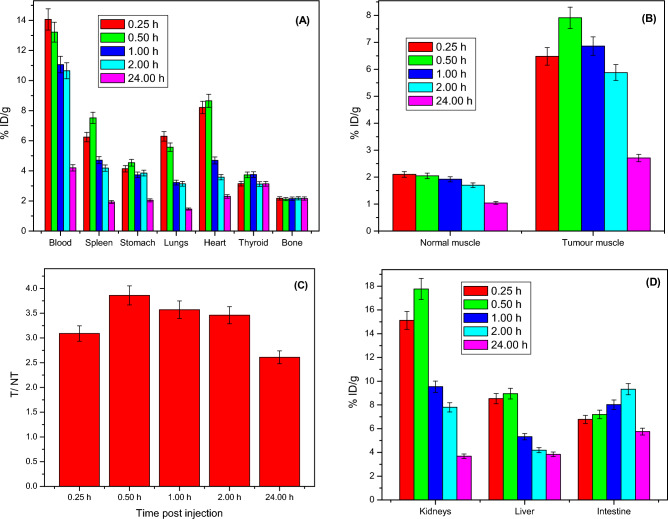
% ID/g organ of the radioiodinated compound 4 in tumour bearing mice (**A**) non-target organs (**B**) % ID/g organ in normal and tumour muscles (**C**) tumour muscles/normal muscles (T NT) (D) excretory organs.

Biodistribution of radioiodinated compound 4 in the animal modes indicates that most organs have no specific accumulation in all non-target organs and showed rapid clearance from soft tissues (Fig. 9A). The targeting ability of radioiodinated compound 4 towards cancer cells was studied in murine-bearing sarcoma. Figure 9B illustrates the tumor uptake showing higher values in all studied time points in a comparison with normal sites. Furthermore, T/NT (target to non-target ratio) was greater than one-fold at all time points, reaching its highest value at one hour post-injection with a value of 3.86 (Fig. 9C). This high ratio (T/NT) indicates that radioiodinated compound 4 can target cancer sites efficiently. The radioiodinated compound 4 was excreted through both urinary and hepatobiliary pathways, this was cleared in Fig. 9D which detected accumulation in the organs of execration (kidneys and liver). (supplementary file).

## Conclusion

Our strategy for the synthesis of ethyl-6-amino-5-cyano-2-methyl-4-((1S,2R,3R,4R)-1,2,3,4,5-pentahydroxypentyl) nicotinate **1** was simply through a multicomponent reaction of D-glucose, malononitrile and ethyl acetoacetate in the presence of ammonium acetate. Then, the preparation of thirteen molecularly designed nicotinate derivatives was performed from the reactions of ethyl-6-amino-5-cyano-2-methyl-4-((1S,2R,3R,4R)-1,2,3,4,5-pentahydroxypentyl) nicotinate 1 with some selected aromatic and heterocyclic amines. All the newly synthesized compounds were prepared by conventional method and under microwave irradiation. The anti-cancer and biological activities of the produced compounds have been evaluated. Also, it was noted that compounds 4 and 8 have stronger cytotoxic activities than other compounds because they have hydrophilic and lipophilic parts. The radioiodinated compound 4 can target the cancer site efficiently. After careful studies, the newly synthesized compounds may be promising candidates as novel cancer theranostic agents.

### Supplementary Information

Supplementary Information.

## Data Availability

All data generated or analyzed during this study are included in this published article.
