# Supplementary material for: Novel pyridine bearing pentose moiety-based anticancer agents: design, synthesis, radioiodination and bioassessments
Source: Sci Rep. 2024 Feb 1;14:2738. doi: 10.1038/s41598-024-53228-4 (PMC10834463; doi:10.1038/s41598-024-53228-4)
Supplement: Supplementary file 1 — Supplementary Information. [file 41598_2024_53228_MOESM1_ESM.pdf]

# 1- $^{1}$H-NMR

**Supplementary figures legends:**


| | |
|---|---|
| **Suppl. Fig. 1** | $^{1}$H-NMR of compound 1 |
| **Suppl. Fig. 2** | $^{1}$H-NMR of compound 2 |
| **Suppl. Fig. 3** | $^{1}$H-NMR of compound 3 |
| **Suppl. Fig. 4** | $^{1}$H-NMR of compound 4 |
| **Suppl. Fig. 5** | $^{1}$H-NMR of compound 5 |
| **Suppl. Fig. 6** | $^{1}$H-NMR of compound 6 |
| **Suppl. Fig. 7** | $^{1}$H-NMR of compound 7 |
| **Suppl. Fig. 8** | $^{1}$H-NMR of compound 8 |
| **Suppl. Fig. 9** | $^{1}$H-NMR of compound 9 |
| **Suppl. Fig. 10** | $^{1}$H-NMR of compound 10 |
| **Suppl. Fig. 11** | $^{1}$H-NMR of compound 11 |
| **Suppl. Fig. 12** | $^{1}$H-NMR of compound 12 |


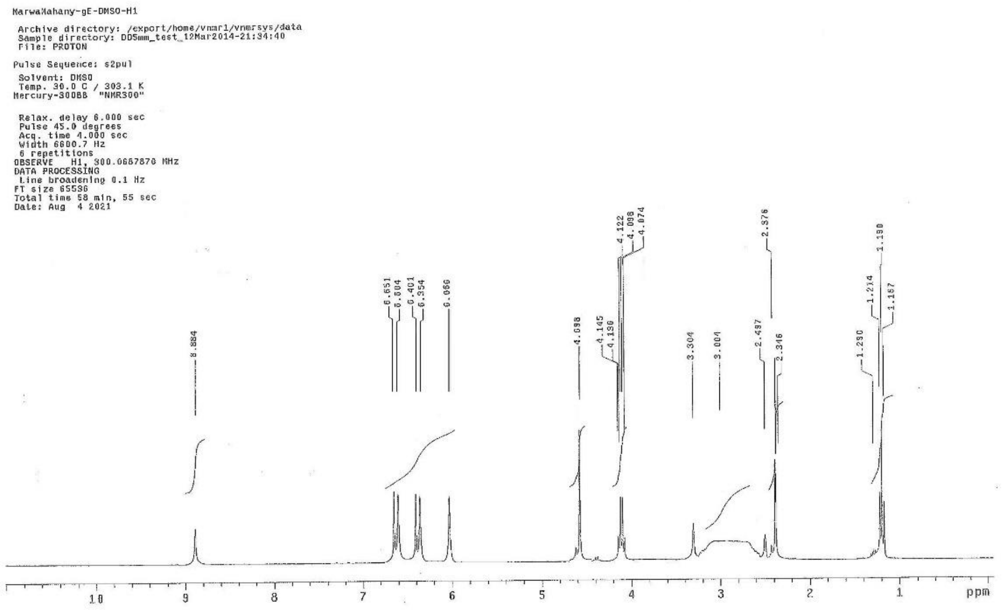


**Suppl. Fig. 1** $^{1}$H-NMR of compound 1

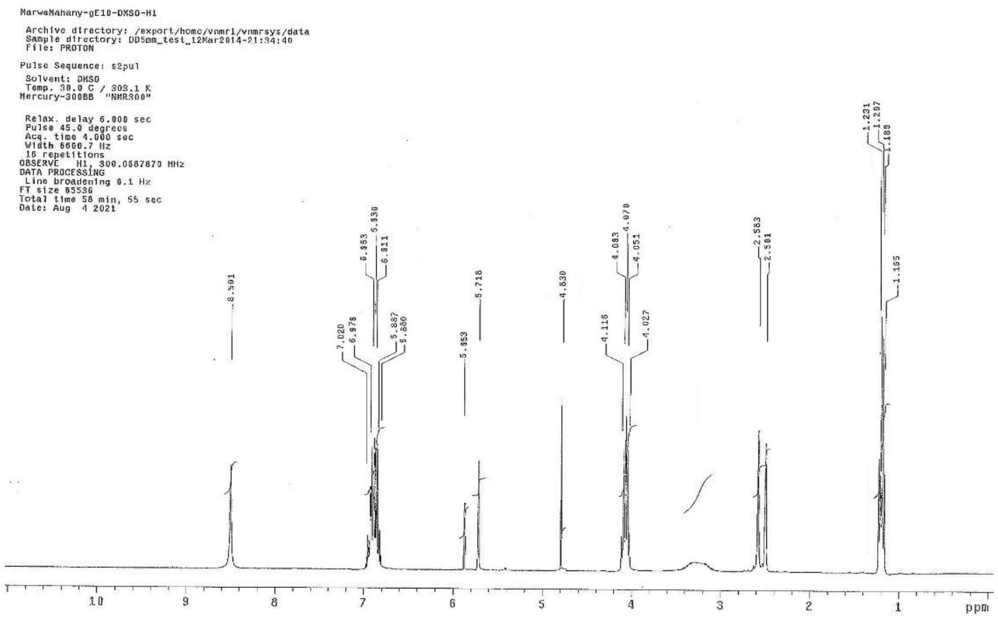


**Suppl. Fig. 2** $^{1}$H-NMR of compound 2

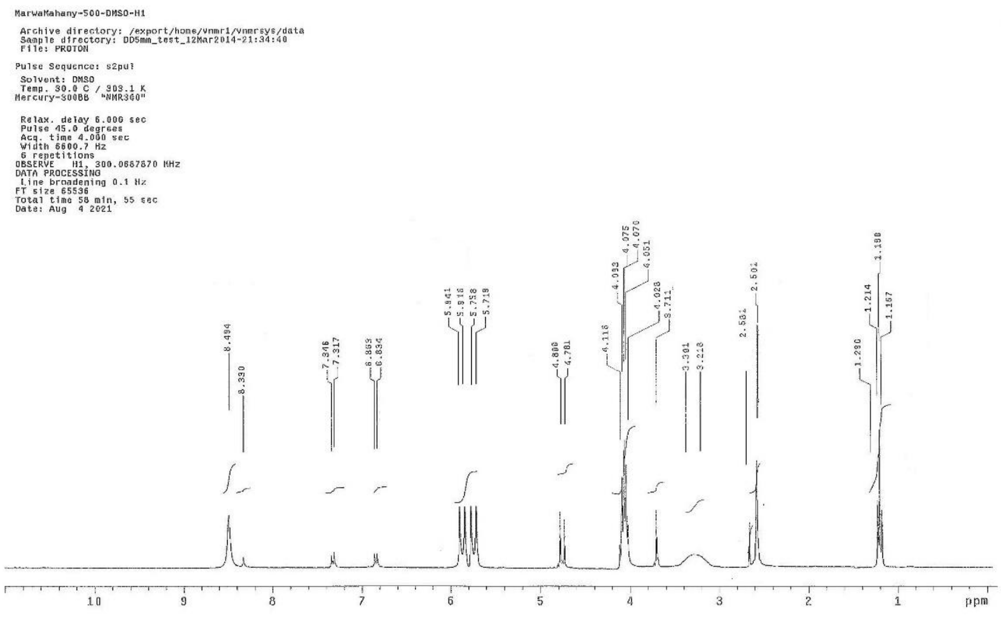


**Suppl. Fig. 3** $^{1}$H-NMR of compound 3

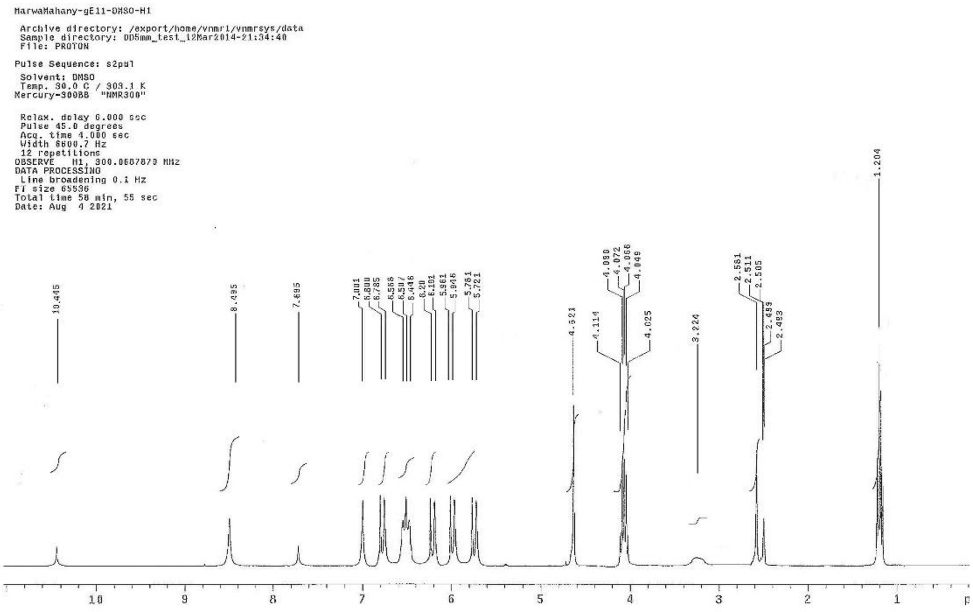


**Suppl. Fig. 4** $^{1}$H-NMR of compound 4

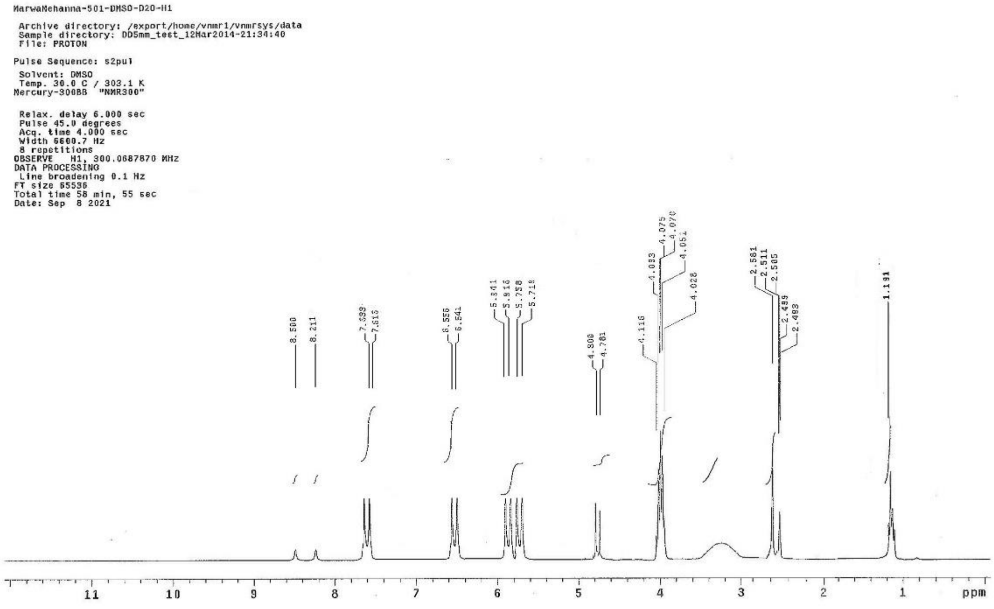

**Suppl. Fig. 5** $^{1}$H-NMR of compound 5

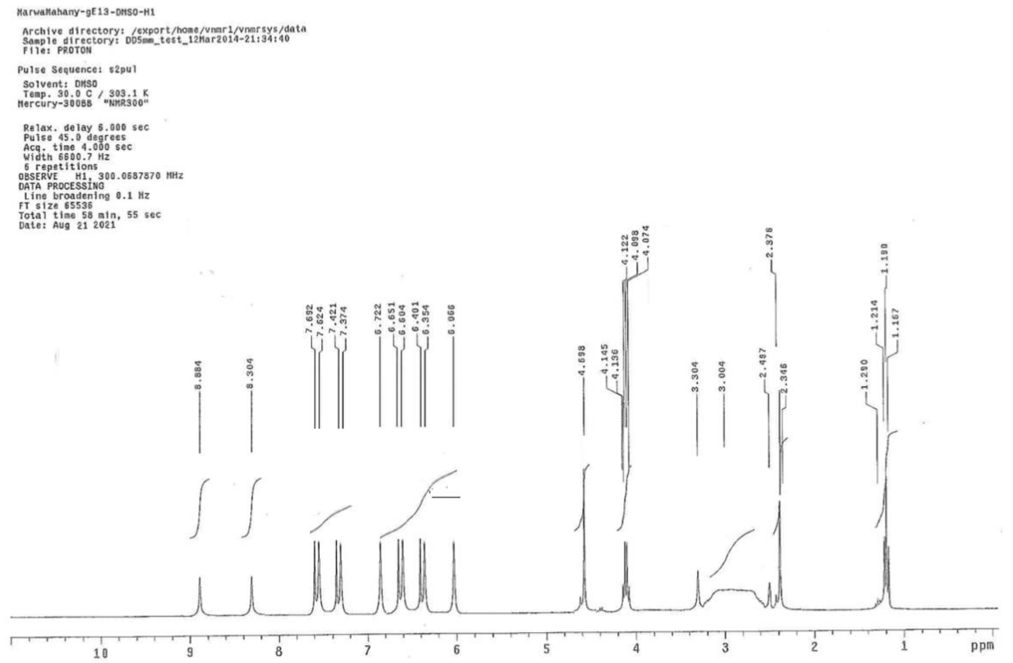


**Suppl. Fig. 6** $^{1}$H-NMR of compound 6

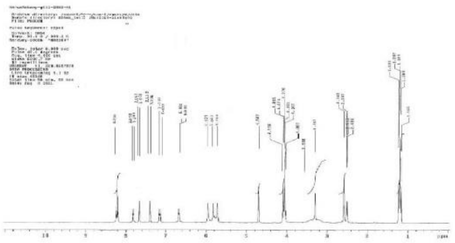

**Suppl. Fig. 7** $^{1}$H-NMR of compound 7

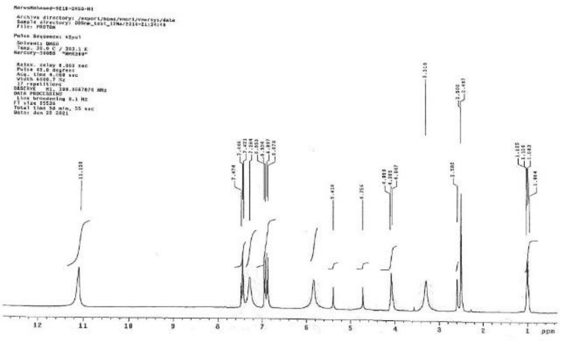

**Suppl. Fig. 8** $^{1}$H-NMR of compound 8

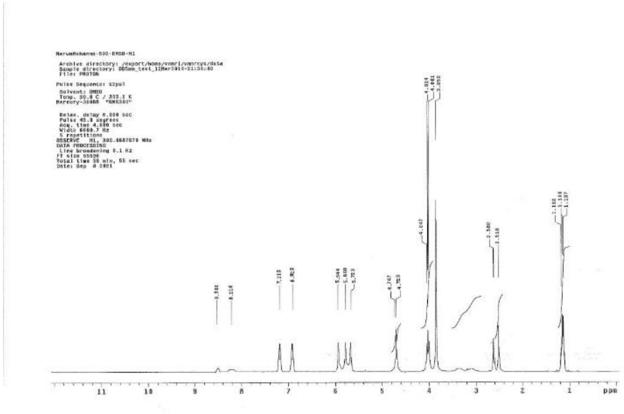


**Suppl. Fig. 9** $^{1}$H-NMR of compound 9

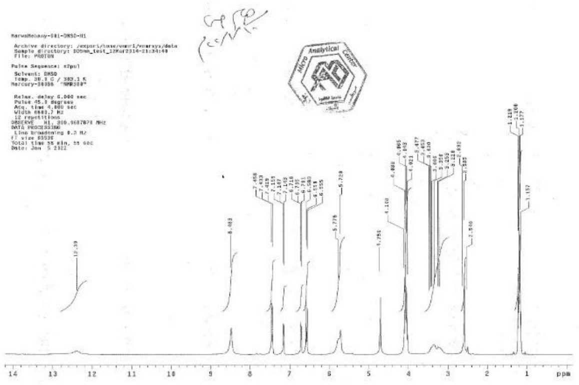

**Suppl. Fig. 10** $^{1}$H-NMR of compound 10

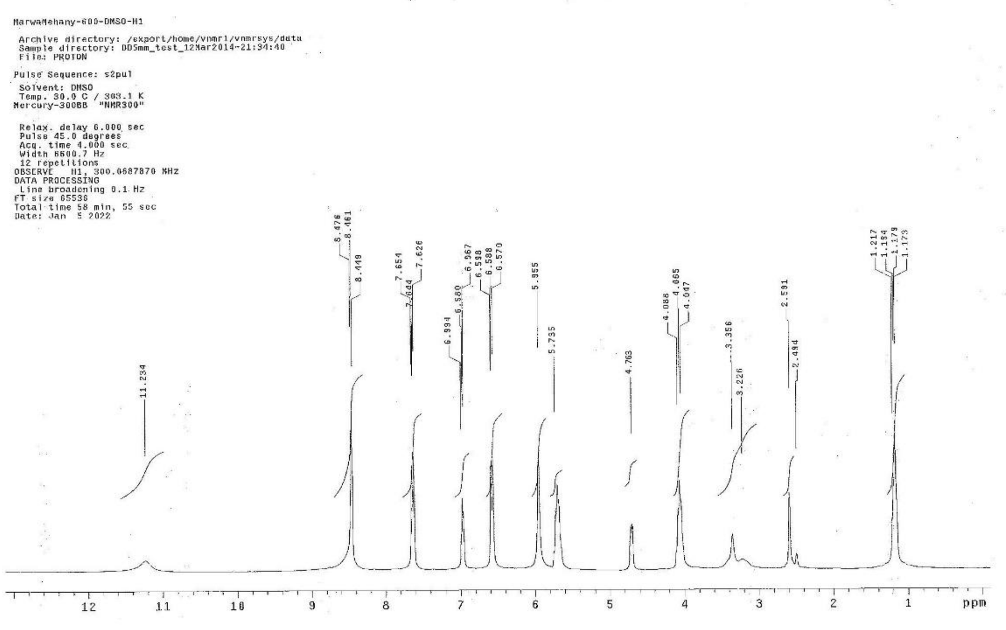

**Suppl. Fig. 11** $^{1}$H-NMR of compound 11

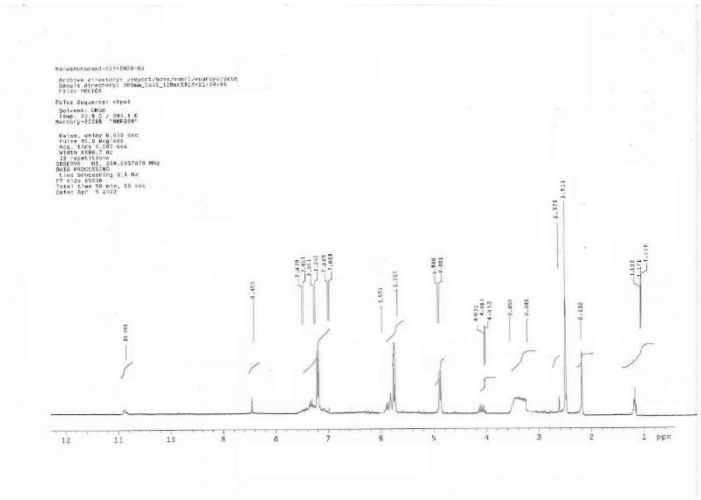


**Suppl. Fig. 12** $^{1}$H-NMR of compound 12

# 2- <u>Radioiodination study:</u>

**a- CAT factor**

| CAT (μg) | Free | Labeled |
|---|---|---|
| **100** | 36.6 | 63.4 |
| **200** | 29.5 | 70.5 |
| **300** | 17.78 | 82.22 |
| **400** | 10.78 | 89.22 |
| **500** | 4.41 | 95.59 |
| **600** | 13.8 | 86.2 |

**b- pH factor**

| pH | Free | Labeled |
|---|---|---|
| **4** | 34.4 | 65.6 |
| **5** | 21.9 | 78.1 |
| **6** | 15.9 | 84.1 |
| **7** | 8.26 | 91.74 |
| **8** | 4.41 | 95.59 |
| **9** | 25 | 75 |

**c- Substrate factor**

| Sub. (μg) | Free | Labeled |
|---|---|---|
| **100** | 44.4 | 55.6 |
| **200** | 36 | 64 |
| **300** | 9.26 | 90.74 |
| **400** | 4.41 | 95.59 |
| **500** | 4.41 | 95.59 |
| **600** | 4.41 | 95.59 |

**d- Time factor**

| Time, min | Free | Labeled |
|---|---|---|
| **15** | 29.3 | 70.7 |
| **30** | 4.41 | 95.59 |
| **60** | 4.41 | 95.59 |
| **90** | 4.41 | 95.59 |
| **120** | 4.41 | 95.59 |

| | | |
|---|---|---|
| **360** | 4.41 | 95.59 |
| **24 h** | 4.41 | 95.59 |

# 3- Biodistribution study:

| **% Injected dose/gram organ at different time post injection** | | | | | |
|---|---|---|---|---|---|
| **Organs** | **0.25 h** | **0.5** | **1 h** | **2 h** | **24 h** |
| **Blood** | **14.06** | **13.21** | **11.06** | **10.65** | **4.20** |
| **Kidneys** | **15.12** | **17.77** | **9.53** | **7.8** | **3.68** |
| **Liver** | **8.53** | **8.95** | **5.32** | **4.19** | **3.84** |
| **Spleen** | **6.24** | **7.52** | **4.71** | **4.18** | **1.93** |
| **Intestine** | **6.78** | **7.20** | **8.02** | **9.33** | **5.75** |
| **Stomach** | **4.14** | **4.54** | **3.73** | **3.85** | **2.04** |
| **Lungs** | **6.29** | **5.57** | **3.22** | **3.14** | **1.46** |
| **Heart** | **8.21** | **8.65** | **4.69** | **3.58** | **2.30** |
| **Thyroid** | **3.15** | **3.73** | **3.75** | **3.12** | **3.14** |
| **Muscle** | **2.10** | **2.05** | **1.92** | **1.70** | **1.04** |
| **Bone** | **2.17** | **2.12** | **2.15** | **2.18** | **2.16** |
| **Tumour** | **6.48** | **7.91** | **6.86** | **5.88** | **2.71** |
| **T/NT** | **3.09** | **3.86** | **3.57** | **3.46** | **2.61** |
